# Efficacy and safety of conventional transarterial chemoembolization for hepatocellular carcinoma using a glass membrane emulsification device: comparison with a three-way stopcock

**DOI:** 10.1007/s11604-026-01995-7

**Published:** 2026-05-06

**Authors:** Yusuke Ono, Yasushi Kimura, Yu Masuda, Hiroki Satomura, Kosuke Tomotake, Yuji Koretsune, Hiroki Yano, Daisuke Katayama, Kaishu Tanaka, Hiroki Higashihara, Noriyuki Tomiyama

**Affiliations:** 1 The Department of Diagnostic and Interventional Radiology, The University of Osaka Graduate School of Medicine, 2-2 Yamadaoka, Suita, Osaka Japan; 2 The Department of High Precision Image-guided Percutaneous Intervention, The University of Osaka Graduate School of Medicine, 2-2 Yamadaoka, Suita, Osaka Japan

**Keywords:** Hepatocellular carcinoma, Chemoembolization, Lipiodol emulsion, Glass membrane emulsification device, Recurrence-free survival

## Abstract

**Purpose:**

To compare the efficacy and safety of conventional transarterial chemoembolization (cTACE) using a glass membrane emulsification device (GMD) versus a three-way stopcock (3WS) in treatment-naïve target lesions of hepatocellular carcinoma (HCC).

**Materials and methods:**

We retrospectively analyzed 54 patients (73 target lesions) treated with GMD-cTACE and 39 patients (55 target lesions) treated with 3WS-cTACE. The emulsion consisted of 5 mL of Lipiodol and 2.5 mL of an iodinated nonionic contrast medium (iomeprol 300 mg I/mL) containing 50 mg of epirubicin. The primary endpoints were the direct treatment effect on target lesions, assessed by RECICL (2015). Recurrence-free survival (RFS), safety and modified albumin–bilirubin (mALBI) grade changes were evaluated as secondary endpoints.

**Results:**

TE4 rates were 86.3% in the GMD group and 76.4% in the 3WS group (*P* = 0.15). The combined TE4 + TE3 rates were 93.2% versus 83.6%, respectively (*P* = 0.09). The median RFS of target lesions was 1,047 days in the GMD group and 362 days in the 3WS group. RFS of target lesions tended to be longer with GMD (log-rank *P* = 0.058), with 1-year RFS estimates of 0.740 (GMD) and 0.470 (3WS). Restricted mean survival time (RMST) up to 365 days favored GMD (difference, 40.2 days; 95% CI, − 0.8 to 81.1; *P* = 0.054). Changes in hepatic reserve were not significantly different between groups.

**Conclusion:**

GMD-cTACE showed numerically longer RFS of target lesions than 3WS-cTACE, with no significant between-group difference in hepatic function deterioration. Standardizing emulsion quality with GMD may improve the durability of local control in cTACE; larger prospective studies are warranted.

**Supplementary Information:**

The online version contains supplementary material available at 10.1007/s11604-026-01995-7.

## Introduction

Conventional transarterial chemoembolization (cTACE), first developed in Japan in the 1980s, has become a cornerstone locoregional therapy for unresectable hepatocellular carcinoma (HCC) [1, 2]. In current practice, both cTACE and drug-eluting bead TACE (DEB-TACE) are used, with selection guided by appropriate indications and patient-specific factors [3]. cTACE—delivery of a cytotoxic agent emulsified with iodized oil (Lipiodol) followed by embolization with particulate agents—remains the most widely performed TACE technique worldwide. Contemporary guidelines position TACE as the first-line treatment for intermediate-stage (Barcelona Clinic Liver Cancer stage B) HCC [4, 5].

Although several technical factors have been reported to influence the therapeutic effects of cTACE, the therapeutic performance of cTACE is influenced by the physicochemical profile of the Lipiodol–drug emulsion, including emulsion type (water-in-oil (W/O) vs. oil-in-water (O/W)), droplet size, stability, and drug-release kinetics [6]. Manual three-way stopcock (3WS) mixing can yield variable emulsion quality, potentially affecting intratumoral delivery and durability of response [7, 8].

**Fig. 1:**
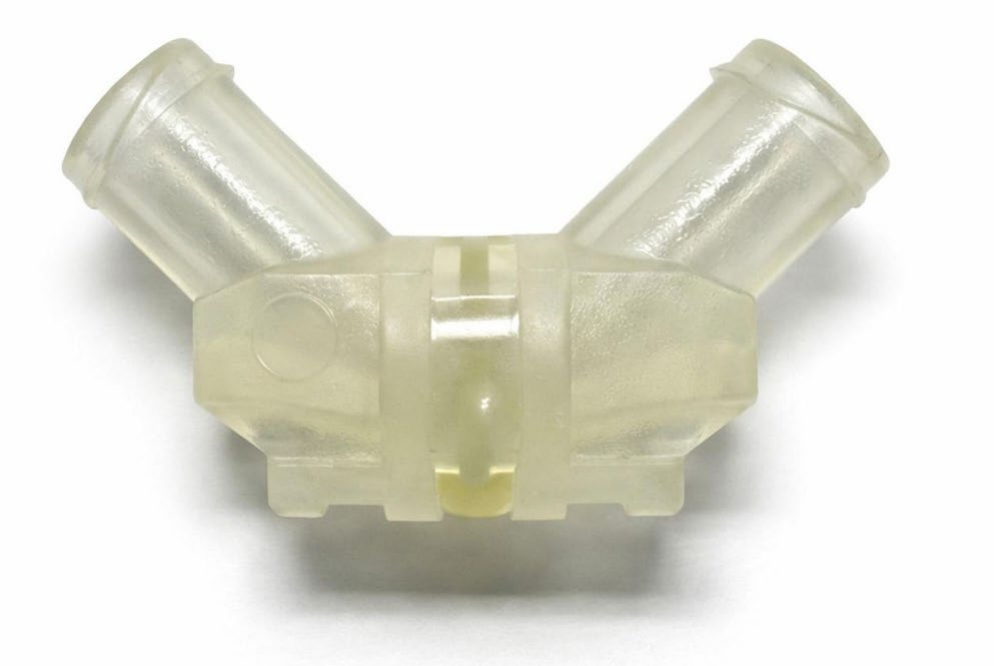
Photograph of the glass-membrane device (GMD) used for emulsion preparation (MicroMagic; Piolax Medical Devices, Inc., Yokohama, Kanagawa, Japan)

To standardize emulsion properties, a glass membrane emulsification device (GMD; MicroMagic; Piolax Medical Devices, Inc., Yokohama, Kanagawa, Japan) was developed (Fig. 1). The device is a connector containing a surface-modified porous glass membrane with pores of approximately 100 μm and two syringe adapters arranged at a 90° angle, which allows easy back-and-forth pumping of the Lipiodol and aqueous drug solution through the membrane. This design enables reproducible formation of uniform W/O emulsions. Preclinical studies have demonstrated that GMD-generated emulsions are highly stable, nearly pure W/O systems with uniform droplet size and slower drug release compared with conventional 3WS mixing, and animal experiments have shown improved intratumoral drug retention with reduced systemic exposure [9–11]. Early clinical reports suggest the feasibility and safety of GMD-assisted cTACE with encouraging local control, although comparative evidence remains limited [12–14].

Given this background, we retrospectively compared cTACE using GMD with cTACE using 3WS in treatment-naïve target lesions of HCC, evaluating tumor response, recurrence-free survival, adverse events, and hepatic function.

## Materials & methods

### Study design and participants

A retrospective study was conducted at a single institution. From October 2019 to September 2022, the study included 54 patients with treatment-naïve target lesions of HCC who underwent cTACE using GMD. Thirty-nine patients with treatment-naïve target lesions of HCC who underwent cTACE using 3WS from January 2017 to September 2019 were included as the historical control group. Only patients who underwent their first cTACE session during the study period were eligible; when multiple cTACE sessions were performed, only the first session within the study period was analyzed. Patients were required to have evaluable contrast-enhanced CT or MRI obtained both before and after the index cTACE for viability assessment, and those lacking adequate pre- and post-treatment imaging or without a target lesion ≥ 1 cm on baseline imaging were excluded. Patients who underwent both techniques during the study period were excluded from the cohorts to avoid duplication. Patients with Child–Pugh class C liver disease or those who had received other treatments (e.g., radiofrequency ablation, surgery, or radiotherapy) within 3 months before or after the initial cTACE were excluded. The study protocol was approved by the Ethics Committee of our Hospital.

In this study, target lesions were considered treatment-naïve on a lesion basis, defined as HCC nodules without any prior locoregional or surgical treatment to that lesion; patients could have received prior HCC treatments for other lesions (e.g., prior hepatectomy), provided that the index target lesion(s) treated with cTACE in the current study had not been previously treated.

### TACE procedure

The procedural workflow, embolic materials, epirubicin dose, and imaging guidance protocol were consistent throughout the study period; the only intended difference between groups was the emulsion-preparation device (GMD vs. 3WS).

Under local anesthesia, cTACE was performed via a femoral approach. We first obtained CT arterial portography from the superior mesenteric artery. We then performed catheter angiography of the celiac trunk and hepatic artery, followed by CT angiography from the proper or common hepatic artery. These studies visualized intrahepatic tumor localization and feeding arteries. Then, we inserted a microcatheter into the feeding artery as selectively as possible. A 1.7-Fr tip microcatheter was typically used, with the specific model selected according to operator preference. Super-selective catheterization was attempted whenever feasible; however, in cases with extensive multifocal disease or when super-selective access to each tumor feeder was not technically achievable, selective catheterization at the segmental/subsegmental level was performed at the operator’s discretion.

For both groups, the emulsion consisted of 5 mL of iodized oil (Lipiodol; Guerbet Japan, Tokyo, Japan) and 50 mg of epirubicin hydrochloride (Nippon Kayaku Co., Ltd., Tokyo, Japan) dissolved in 2.5 mL of a nonionic contrast medium (iomeprol 300 mg I/mL; Iomeron 300; Bracco Japan Co., Ltd., Tokyo, Japan), an aqueous-to-Lipiodol ratio of 1:2. At our institution, the Lipiodol volume was standardized according to the epirubicin dose (1 mL per 10 mg epirubicin). A maximum Lipiodol volume of 10 mL per TACE session was set in adult patients. The emulsion was prepared using either the GMD or a 3WS. In both methods, the Lipiodol and aqueous phase were emulsified by alternately pumping the two syringes through the device ≥ 20 times in each direction (≥ 40 pumping strokes in total) to form a homogeneous emulsion. The emulsion was infused until tumor-selective accumulation of the Lipiodol emulsion was visible on fluoroscopy. Subsequently, 1-mm square gelatin sponge particles (Gelpart; Nippon Kayaku Co., Ltd., Tokyo, Japan), suspended in a nonionic contrast medium, were injected until near stasis of antegrade flow in the tumor-feeding artery was achieved.

### Study outcomes

Tumor response was evaluated using contrast-enhanced computed tomography (CT) or magnetic resonance imaging (MRI) scans before and after treatment. Contrast-enhanced CT was performed as a dynamic multiphasic study, typically including arterial, portal venous, and delayed phases. MRI was predominantly performed as gadoxetic acid–enhanced MRI (EOB-MRI), including T2-weighted imaging, T1-weighted imaging, diffusion-weighted imaging, dynamic contrast-enhanced phases, and hepatobiliary phase imaging. Target lesions were prespecified as up to two lesions —the largest and second-largest tumors— with a longest diameter ≥ 1 cm on baseline imaging. The treatment effect for target lesions was evaluated using the Response Evaluation Criteria in Cancer of the Liver (RECICL, 2015) [15], based on CT or MRI images obtained approximately three months after TACE. Follow-up contrast-enhanced CT or MRI was scheduled at approximately 3 months after cTACE, and thereafter at 3–6-month intervals according to the treating physician’s discretion, or earlier when recurrence was suspected. Recurrence-free survival (RFS) of target lesions was defined as the time from cTACE to loss of TE4 (complete response) on follow-up imaging; lesions without loss of TE4 were censored at the last follow-up. Adverse events after TACE were evaluated using CTCAE v4.0. Changes in modified albumin–bilirubin (mALBI) grade were calculated from laboratory data obtained approximately 3 months after TACE. Tumor location was categorized as central or peripheral according to Asano et al.: tumors within 1 cm of the main portal vein trunk or its first branch were defined as central, and others as peripheral [16].

Time to next treatment was defined as the time from cTACE to initiation of any subsequent HCC treatment after the index cTACE (including treatment for other lesions); patients without subsequent treatment were censored at the last follow-up.

### Statistical analysis

Continuous variables are presented as mean ± standard deviation or median (interquartile range), as appropriate, and were compared using the Student’s t test or the Mann–Whitney U test. Categorical variables are presented as counts (percentages) and were compared using the chi-square test or Fisher’s exact test, as appropriate. Recurrence-free survival (RFS) was analyzed using the Kaplan–Meier method and compared between groups using the log-rank test. Hazard ratios (HRs) with 95% confidence intervals (CIs) were estimated using Cox proportional hazards models. The proportional hazards assumption was assessed using Schoenfeld residuals. In addition, we estimated the restricted mean survival time (RMST) up to 365 days (τ = 365) as a complementary measure of between-group differences in recurrence-free time within the first year. As a sensitivity analysis, we performed a landmark analysis among target lesions achieving TE4 at the first follow-up evaluation (approximately 3 months after cTACE). Time zero was defined as the date of the first follow-up contrast-enhanced CT/MRI, and subsequent local recurrence (loss of TE4) was analyzed using the Kaplan–Meier method and the log-rank test. All statistical analyses were performed using EZR (Saitama Medical Center, Jichi Medical University), a graphical user interface for R (The R Foundation for Statistical Computing, Vienna, Austria). A two-sided *P* value < 0.05 was considered statistically significant.

## Results

### Patient background

The baseline characteristics and follow-up assessments are shown in Table 1. The first response assessment was performed at 85.6 ± 38.4 days in the GMD group and 90.1 ± 36.6 days in the 3WS group (*P* = 0.57). At the first assessment, contrast-enhanced CT was used in 49 patients in the GMD group and 30 patients in the 3WS group, whereas MRI was used in 5 and 9 patients, respectively (*P* = 0.07). There were no significant differences in background characteristics between patients in the two groups.

**Table 1:** Baseline characteristics and follow-up assessment

Variables	GMD group *n* = 54	3WS group *n* = 39	*P *value
*Sex, n (%)*			
Male	41 (75.9)	26 (66.7)	0.33
Female	13 (24.1)	13 (33.3)	
Age (y)	75.8 ± 12.0	75.5 ± 9.1	0.91
*History of prior HCC treatment (other lesions), n (%)*			
Yes	44 (81.5)	27 (69.2)	0.17
No	10 (18.5)	12 (30.8)	
*Etiology, n (%)*			
HCV	22 (40.7)	24 (61.5)	0.23
HBV	11 (20.4)	6 (15.4)	
Alcohol	7 (13.0)	2 (5.1)	
Other	14 (25.9)	7 (18.0)	
*Child-Pugh score, n (%)*			
A	51 (94.4)	33 (84.6)	0.11
B	3 (5.6)	6 (15.4)	
*Up-to-7 criteria, n (%)*			
In	48 (88.9)	31 (79.5)	0.21
Out	6 (11.1)	8 (20.5)	
*BCLC, n (%)*			
0	16 (29.6)	7 (18.0)	0.41
A	24 (44.5)	19 (48.7)	
B	14 (25.9)	13 (33.3)	
Maximum tumor size (mm)	19.0 ± 9.3	17.7 ± 9.7	0.52
*Tumor number, n (%)*			
1	25 (46.3)	13 (33.3)	0.65
2	11 (20.4)	9 (23.1)	
3	5 (9.3)	4 (10.3)	
4	6 (11.1)	3 (7.7)	
5	2 (3.7)	3 (7.7)	
≧6	5 (9.3)	7 (17.9)	
*Tumor location (target lesions), n (%)*			
Peripheral	58 (79.5)	41 (74.5)	0.51
Central	15 (20.5)	14 (25.5)	
*Embolization level, n (%)*			
Super-selective	46 (85.2)	34 (87.2)	0.17
Selective	4 (7.4)	5 (12.8)	
Lobar	4 (7.4)	0 (0)	
AST (IU/L)	30.0 ± 14.4	32.2 ± 14.7	0.48
ALT (IU/L)	25.3 ± 17.9	25.1 ± 13.0	0.96
Total bilirubin (mg/dL)	0.72 ± 0.35	0.78 ± 0.43	0.48
Albumin (g/dL)	3.92 ± 0.44	3.78 ± 0.48	0.18
AFP (ng/mL)	4.5 (1–900)	7 (1–19328)	0.18
DCP (mAU/mL)	52 (12–35412)	45 (11–1570)	0.82
Epirubicin dose administered per session (mg)	22.1 ± 13.3	24.6 ± 14.6	0.40
Time to first response assessment (days)	85.6 ± 38.4	90.1 ± 36.6	0.57
*First response assessment modality, n (%)*			
CT	49 (90.7)	30 (76.9)	0.07
MRI	5 (9.3)	9 (23.1)	

HCV hepatitis C virus, HBV hepatitis B virus, BCLC Barcelona Clinic Liver Cancer, AST aspartate aminotransferase, ALT alanine transaminase, AFP alpha fetoprotein, DCP des-gamma-carboxy prothrombin

Age, AST, ALT, Total bilirubin, albumin are expressed as mean ± standard deviation

AFP and DCP are expressed as median (range)

Tumor location is reported per target lesion; all other variables are reported per patient unless otherwise specified

Central tumors were defined as those within 1 cm of the main portal vein trunk or its first branch [16]

Super-selective was defined as embolization performed at a subsegmental or more distal level

The Lipiodol volume is standardized according to the epirubicin dose (1 mL per 10 mg epirubicin)

### Treatment effects

Table 2 shows the treatment effects after TACE in both groups. The TE4 response rate for target lesions was 86.3% (63/73) in the GMD group and 76.4% (42/55) in the 3WS group (*p* = 0.15). The TE4 + TE3 (partial response) rate for target lesions was 93.2% (68/73) in the GMD group and 83.6% (46/55) in the 3WS group (*P* = 0.09).

**Table 2:** Lesion-level treatment effect at the first response assessment based on RECICL 2015

Complete response, *n* (%)	GMD group *n* = 73	3WS group *n* = 55	*P* value
TE4	63 (86.3)	42 (76.4)	0.15
TE1/2/3	10 (13.7)	13 (23.6)	
Objective response, n (%)			
TE3/4	68 (93.2)	46 (83.6)	0.09
TE1/2	5 (6.8)	9 (16.4)	

Data are shown per target lesion at the first post-treatment assessment; treatment effect was evaluated using RECICL 2015

**Fig. 2:**
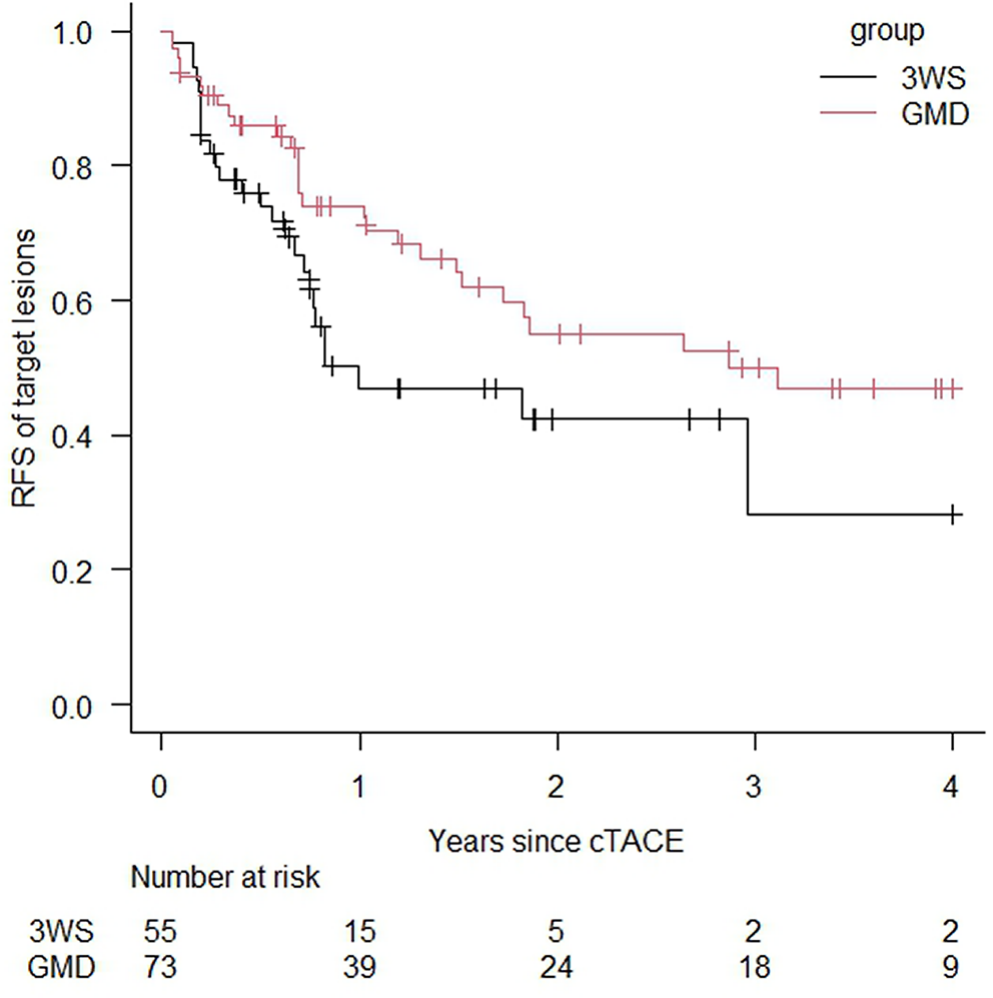
Kaplan–Meier curve of recurrence-free survival (RFS) for target lesions treated with cTACE using a GMD versus a 3WS. Kaplan–Meier analysis showed a higher RFS in the GMD group than in the 3WS group (log-rank *P* = 0.058)

The Kaplan-Meier curve of RFS of target lesions is shown in Fig. 2. The median RFS was 1,047 days in the GMD group and was 362 days in the 3WS group. Kaplan–Meier analysis showed a higher RFS in the GMD group than in the 3WS group (*P* = 0.058). The Kaplan–Meier–estimated 1-year RFS was 0.740 in the GMD group and 0.470 in the 3WS group. The restricted mean RFS time up to 365 days (τ = 365) was longer in the GMD group than in the 3WS group (RMST difference, 40.2 days; 95% CI, − 0.8 to 81.1; *P* = 0.054). The proportional hazards assumption was not violated (Schoenfeld residual test, *P* = 0.76). In the landmark cohort, the Kaplan–Meier–estimated 1-year RFS of target lesions was 0.756 in the GMD group and 0.623 in the 3WS group, with no statistically significant difference (log-rank *P* = 0.21). These findings were directionally consistent with the primary analysis, although the between-group difference did not reach statistical significance (Online Resource 1). Time to next treatment (including treatment for other lesions) tended to be longer in the GMD group, although the difference was not statistically significant (*P* = 0.23) (Online Resource 2).

### Adverse events

Table 3 shows the adverse events after TACE in both groups. Grade ≥ 3 elevations in AST and ALT (per CTCAE v4.0) occurred in 23/54 (42.6%) and 16/54 (29.6%) patients in the GMD group and in 11/39 (28.2%) and 6/39 (15.4%) patients in the 3WS group, respectively (*P* = 0.16 and *P* = 0.11). There were no significant differences in serum bilirubin and albumin levels between the two groups. Nausea/vomiting were more frequent in the GMD group, whereas fatigue was more frequent in the 3WS group; all were Grade ≤ 2.

**Table 3:** Adverse events after TACE

Blood test (≧G3), *n* (%)	GMD group *n* = 54	3WS group *n* = 39	*P* value
AST	23 (42.6)	11 (28.2)	0.16
ALT	16 (29.6)	6 (15.4)	0.11
T-bil	0 (0)	0 (0)	–
Alb	0 (0)	0 (0)	–
Clinical sign (≧G1), n (%)			
Stomachache	15 (27.8)	10 (25.6)	0.82
Fever	15 (27.8)	14 (35.9)	0.4
Nausea	19 (35.2)	7 (17.9)	0.07
Vomiting	9 (16.7)	2 (5.1)	0.09
Weariness	12 (22.2)	16 (41.0)	0.05

### Changes in mALBI grade

Table 4 shows the changes in mALBI grade three months after TACE in both groups. The number of patients with improvement, no change, or deterioration of mALBI grade after TACE was 7 (13.0%), 41 (75.9%), and 6 (11.1%) in the GMD group and 7 (17.9%), 26 (66.7%), and 6 (15.4%) in the 3WS group, respectively. There was no significant difference between the two groups (*P* = 0.62).

**Table 4:** Changes in mALBI grade three months after TACE

Changes, *n* (%)	GMD group *n* = 54	3WS group *n* = 39	*P* value
Improvement	7 (13.0)	7 (17.9)	0.62
No change	41 (75.9)	26 (66.7)	
Deterioration	6 (11.1)	6 (15.4)	

## Discussion

In this single-center retrospective comparison of cTACE performed with a GMD versus a 3WS, early radiologic responses were broadly comparable between groups. RFS of target lesions tended to be longer with GMD (log-rank *P* = 0.058), with higher 1-year RFS estimates (0.740 vs. 0.470) and a longer RMST within 365 days (difference, 40.2 days; *P* = 0.054). In the landmark sensitivity analysis restricted to lesions achieving TE4 at first follow-up, RFS remained numerically higher with GMD but was not statistically significant (log-rank *P* = 0.212). Elevations in hepatic enzymes were numerically more frequent after GMD-cTACE, but there were no significant differences in severe adverse events or in deterioration of hepatic reserve as assessed by mALBI. Taken together, these results suggest a potential durability advantage with GMD-prepared emulsions, although the study may have been underpowered to detect statistically significant differences.

Superior 1-year local tumor control with GMD is consistent with previous preclinical evidence showing that emulsion type, droplet size, and stability govern intratumoral drug delivery in cTACE. In vitro formulation studies demonstrated that GMDs produce nearly 100% W/O emulsions with uniform droplets and markedly slower drug release than 3WS mixing, across multiple agents (e.g., epirubicin, cisplatin) [9, 10]. In a rabbit VX2 tumor model, GMD-cTACE achieved higher intratumoral drug concentrations with lower systemic exposure and greater tumor necrosis than conventional emulsions [11]. Early clinical series of GMD-assisted cTACE have reported favorable local control without excess toxicity. In a single-arm study of 26 patients, the first imaging assessment (median 83 days) showed an objective response in all evaluated cases (CR 90%, PR 10%), with local recurrence rates of 24.2% at 200 days and 49.4% at 300 days [12]. In patients with solitary HCC, local control after GMD-TACE was 100% at 6 months and 81.8% at 1 year, and no serious complications were observed in the GMD-TACE group (*n* = 22) [13]. In a comparative cohort (non-GMD, *n* = 27; GMD, *n* = 44), local recurrence was 0.0% at 12 months in the GMD group versus 16.7% in the non-GMD (3WS) group (log-rank *P* < 0.05) [14]. Taken together, these reports are consistent with the concept that emulsion quality may influence the durability of local tumor control after cTACE. Mechanistically, GMD preparation has been shown to produce a more stable, W/O-dominant emulsion with smaller and more uniform droplets than conventional 3WS mixing [7, 8, 17]. A stable W/O emulsion is expected to traverse tumor-feeding arterioles more effectively, achieve more homogeneous intratumoral Lipiodol deposition, and sustain local drug availability through slower drug elution [18, 19]. In this context, our observed pattern appears mechanistically coherent: although early response rates were similar, the Kaplan–Meier–estimated 1-year RFS was higher in the GMD group (0.740 vs. 0.470), the overall log-rank test showed a trend favoring GMD (*P* = 0.058), and RMST up to 365 days favored GMD (difference, 40.2 days; *P* = 0.054). These findings are hypothesis-generating and should be interpreted cautiously given the retrospective, nonrandomized design.

Although initial TE4 (and TE3 + TE4) rates did not differ significantly, this may reflect the fact that a predominantly super-selective approach, when technically feasible, together with adjunct gelatin sponge embolization can achieve substantial early ischemic necrosis in both groups, thereby attenuating detectable differences at the first assessment. In contrast, recurrence over subsequent months may better capture formulation-dependent pharmacokinetics: drug-release kinetics and emulsion stability are likely to influence residual microscopic disease and early regrowth. Notably, despite the 1:2 aqueous-to-Lipiodol ratio favoring a W/O state, 3WS mixing can still yield heterogeneous emulsions with O/W components, whereas GMD maintains a nearly 100% W/O state, which may contribute to greater durability of TE4 status [9, 10]. Preclinical studies have shown that W/O Lipiodol emulsions achieve greater tumor targeting and retention than O/W emulsions, with preferential lodgment within the tumor microcirculation and enhanced intratumoral uptake of iodized oil–based formulations [20, 21]. Fluid-mechanics simulations further suggest that W/O-dominant emulsions are more prone to microvascular stagnation and distal embolic persistence under conditions relevant to hepatic microcirculation, thereby prolonging local residence time [22]. Together with the established role of Lipiodol as a carrier that sustains intratumoral drug exposure, these data provide a mechanistic rationale for why achieving a nearly 100% W/O, stable emulsion may improve durability of tumor control compared with less stable emulsions containing substantial O/W components [23].

Numerically higher AST/ALT rises after GMD may reflect denser Lipiodol deposition and stronger ischemic-chemotherapeutic intensity, a trade-off commonly seen when embolization is effective. Importantly, we did not observe significant between-group differences in serious adverse events or mALBI worsening, suggesting that in the context of selective catheterization and standardized aftercare, the incremental “on-target” intensity with GMD remains clinically manageable. These findings mirror prior clinical series where GMD-TACE achieved high local control without excess toxicity [12–14]. Given the growing interest in combining TACE with immune checkpoint inhibitors (ICIs), facilitating tumor antigen release through ischemic-chemotherapeutic intensity is important [24, 25]. This concept has been highlighted in recent reviews of systemic–locoregional combination strategies, where performing TACE between courses of ICI-based therapy is proposed to reduce tumor burden and potentially enhance antitumor immunity via tumor antigen release [25]. Although speculative, this potential synergy between GMD-assisted TACE and immunotherapy merits further investigation in prospective settings.

Randomized and prospective data outside GMD have already shown that emulsion stability (preferably W/O) correlates with better pharmacokinetics and treatment performance [8]. Our results extend this concept by demonstrating, in routine practice, that a device designed to enforce W/O dominance and droplet uniformity can translate into lower 1-year recurrence, aligning with early GMD clinical experiences [12–14] and the broader IR literature emphasizing emulsion physics as a determinant of cTACE efficacy [7, 8]. Historically, cTACE—pioneered in Japan in the 1980s and now the mainstay for BCLC-B—has thrived through incremental refinements in technique and materials [1–5]. GMD is best viewed as the next refinement: quality-assured emulsion as a new standard input to a mature therapy.

This analysis has several limitations. First, the sample size is modest, limiting statistical power and the robustness of subgroup analyses (e.g., tumor size, segmental location, vascularity). Second, the retrospective, nonrandomized design introduces selection bias and unmeasured confounders (e.g., subtle differences in tumor biology, comorbidity, or peri-procedural care). Given the limited sample size, propensity-score weighting or extensive multivariable adjustment could yield unstable estimates; therefore, residual confounding cannot be excluded. Third, it is a single-center study; operator technique and institutional protocols may limit generalizability. Fourth, the cohorts are non-contemporaneous (3WS: 2017–2019; GMD: 2019–2022), so era effects (learning curve, imaging quality, supportive therapy) cannot be fully excluded. Fifth, the imaging modality at the first response assessment (contrast-enhanced CT vs. MRI) was at the treating physician’s discretion and could not be standardized retrospectively; therefore, modality selection may have influenced recurrence detection. Sixth, the number of patients at risk decreased substantially after 2 years, limiting the robustness of the long-term Kaplan–Meier estimates. Therefore, long-term outcomes should be interpreted with caution.

## Conclusion

Our results support the feasibility and potential clinical benefit of using GMD in cTACE. By providing a more stable and effective emulsion, GMD may improve tumor control without increasing toxicity. Further prospective studies are warranted to validate these findings and establish GMD as a standard device in TACE practice.

Ethics declarations.

## Electronic supplementary material

Below is the link to the electronic supplementary material.

Online Resource 1 (ESM_1.pdf): Kaplan–Meier curves for recurrence-free survival (RFS) of target lesions in the landmark cohort. The landmark cohort included target lesions that achieved TE4 at the first follow-up contrast-enhanced CT/MRI (approximately 3 months after cTACE). Time zero was defined as the date of the first follow-up imaging. One-year RFS estimates were 0.756 in the GMD group and 0.623 in the 3WS group (*P* = 0.21). Tick marks indicate censoring. Numbers at risk are shown below the x-axis.

Online Resource 2 (ESM_2.pdf): Kaplan–Meier curve for next treatment-free survival after cTACE. The event was defined as initiation of any subsequent HCC treatment after the index cTACE (including treatment for other lesions). Patients without subsequent treatment were censored at the last follow-up. Tick marks indicate censoring. Numbers at risk are shown below the x-axis.

## Data Availability

De-identified datasets generated and/or analyzed during the current study are not publicly available due to institutional and legal restrictions on sharing clinical data. However, the data are available from the corresponding author upon reasonable request and with permission from the Institutional Review Board of Osaka University Hospital (IRB #24445).
