# Supplementary figures and images for: Efficacy and safety of conventional transarterial chemoembolization for hepatocellular carcinoma using a glass membrane emulsification device: comparison with a three-way stopcock
Source: Jpn J Radiol. 2026 May 6;44(8):1468–76. doi: 10.1007/s11604-026-01995-7 (PMC13400582; doi:10.1007/s11604-026-01995-7)

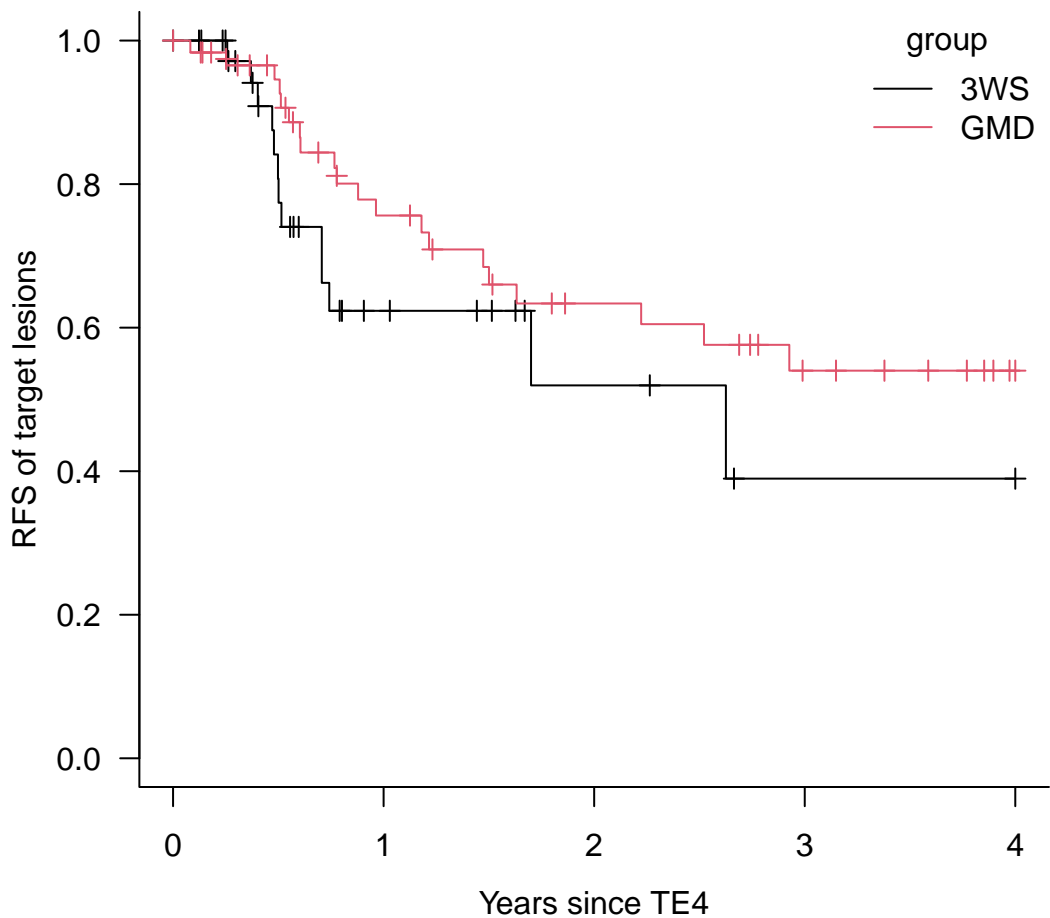

Number at risk

|     |    |    |    |    |   |
|-----|----|----|----|----|---|
| 3WS | 42 | 12 | 5  | 2  | 2 |
| GMD | 63 | 34 | 22 | 14 | 6 |

Supplement: Supplementary file 1 — Online Resource 1 (ESM_1.pdf): Kaplan–Meier curves for recurrence-free survival (RFS) of target lesions in the landmark cohort. The landmark cohort included target lesions that achieved TE4 at the first follow-up contrast-enhanced CT/MRI (approximately 3 months after cTACE). Time zero was defined as the date of the first follow-up imaging. One-year RFS estimates were 0.756 in the GMD group and 0.623 in the 3WS group (P = 0.21). Tick marks indicate censoring. Numbers at risk are shown below the x-axis. [file 11604_2026_1995_MOESM1_ESM.pdf]

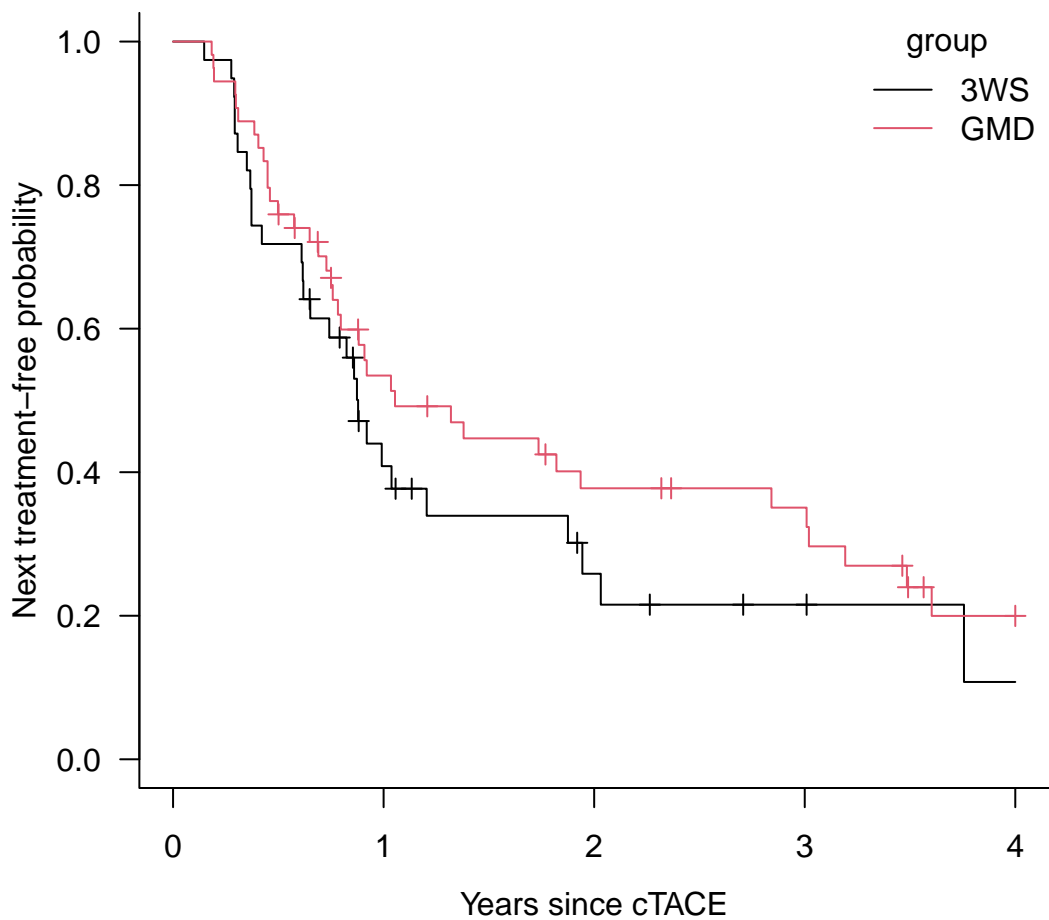

Number at risk

|     |    |    |    |    |   |
|-----|----|----|----|----|---|
| 3WS | 39 | 13 | 6  | 3  | 1 |
| GMD | 54 | 25 | 16 | 13 | 5 |

Supplement: Supplementary file 2 — Online Resource 2 (ESM_2.pdf): Kaplan–Meier curve for next treatment-free survival after cTACE. The event was defined as initiation of any subsequent HCC treatment after the index cTACE (including treatment for other lesions). Patients without subsequent treatment were censored at the last follow-up. Tick marks indicate censoring. Numbers at risk are shown below the x-axis. [file 11604_2026_1995_MOESM2_ESM.pdf]
